# Factors influencing the performance of clinical research networks to improve the success of cancer clinical trials: A scoping review and organizational analysis

**DOI:** 10.1017/cts.2025.10210

**Published:** 2025-12-17

**Authors:** Elizabeth Jane Paton, Tim Luckett, Gerald Blaise Fogarty, Anthony Greville Shannon, Deborah Debono

**Affiliations:** 1 University of Technology Sydney, Faculty of Health, Ultimo, Australia; 2 University of Notre Dame, School of Medicine, Darlinghurst, Australia; 3 Icon Cancer Centre, Sydney, Australia; 4 Sydney Brachytherapy Research Institute, St Leonards, Australia; 5 University of Notre Dame, School of Education, Broadway, Australia; 6 Australian Institute of Technology and Commerce, Haymarket, Australia

**Keywords:** Systematic review, cancer, clinical research networks, randomized controlled trials, clinicalresearch

## Abstract

Cancer clinical research networks (CRNs) play a vital role in medical research globally by generating investigator-initiated research, pooling expertise, and enabling recruitment across multiple sites. Completing clinical trials is challenging. Delays can slow the generation of evidence needed to refine the best patient treatments. The aim of this review was to identify factors that have been either proposed or shown by research to influence the performance of cancer CRNs to improve trial success, outcomes and impact. A scoping review was conducted using a systematic search across five databases [PROSPERO CRD42023414241]. Records were screened for eligibility. For included articles, data on factors and research methods were extracted independently by up to three reviewers, and disagreements resolved by discussion. 1928 articles were returned, 13 were included. Articles reported on 11 membership-based cancer CRNs with headquarters in four countries (eight in the USA). Factors influencing CRN performance broadly fell into six categories: site, CRN, patient, regulatory, policy and industry factors, with subcategories in each case. These findings may help to inform future research to prioritize and improve the day-to-day performance of membership-based cancer CRNs and other trial sponsors to optimize clinical trial success. Further research is warranted.

## Introduction

Evidence-Based Medicine relies on the evidence generated from completed high-quality clinical trials to improve treatments for patients worldwide. However, fewer than one in every 20 cancer patients participate in clinical trials [1–4], about two in every three cancer trials do not recruit sufficient patients [1,5,6]. This slows the generation of evidence needed to improve the standard of care.

Cancer Clinical Research Networks (CRNs) play a vital role in global medical research by generating novel research questions. CRNs are collaborative research groups of practicing clinician-researchers (medical, nursing, and allied health practitioners and career clinical researchers). CRNs undertake investigator-initiated clinical research trials (IITs) in various diseases and that support a (voluntary or paid) membership base. They can be local, national or international, and exist as unincorporated or incorporated legal entities, affiliated with or without a university. Typically, the types of clinically-relevant research generated by CRNs is beyond the scope of commercial trial sponsors (pharmaceutical companies or device manufacturers). CRNs can be described as successful if they generate, conduct, accrue to and complete IIT studies that answer important research questions.

This study aimed to:

1. Identify and describe the original research literature which evaluates the performance of cancer CRNs that conduct cancer clinical trials worldwide and characterize the organizational arrangements of these cancer CRNs,
2. Characterize the eligible articles included in this study,
3. Identify, define and characterize the factors influencing the CRN performance of the clinical trials they conduct, and
4. With consideration to the evidence currently available, identify any knowledge gaps to allow for the identification of future research initiatives.

## Materials and methods

We performed a scoping review using a systematic search strategy [7] across five databases with systematic review of literature to identify the factors that have been used or proposed that influence the performance of cancer CRNs who conduct clinical trials. We conducted a scoping review for several reasons. A systematic review involves a clearly defined topic and question. The examination of factors influencing the performance of CRNs, specifically cancer CRNs, to successfully generate, conduct, accrue to and complete trials is a pluralistic area informed by methodologically diverse research. The scoping method involves review, analysis and synthesis of a broad scope of literature. Unlike systematic reviews, scoping studies do not assess the quality of studies and as they require the literature to be analytically reinterpreted [8]. This method is appropriate given the complexity of the area and the aims of this study, which is to build a comprehensive picture specific to the cancer CRN research landscape, rather than to weigh up the levels of evidence in relation to a specific question (which would be more akin to the approach used in a traditional systematic review).

The protocol was registered with the international prospective register of systematic reviews (PROSPERO CRD42023414241; registered 12 April 2023), in accordance with the Preferred Reporting Items for Systematic Reviews Protocols (PRISMA-P) guidelines. A structured eligibility criteria framework in line with the Population Intervention Comparator Study Characteristics Other (PICOS) framework [9] was applied to the full text reviews (see Table 1).

**Table 1.** Describes the structured eligibility criteria framework (in brief) in line with the PICOS framework

PICOS framework eligibility criteria		
**Criteria**	Inclusion	Exclusion
**Population**	Articles related to CRNs, oncology patients, clinical research and treatments.	Articles containing individual clinical trial results, commercial sponsorship, integrative therapies (e.g., natural and complementary medicine) and non-human research.
**Intervention/ exposure**	Articles evaluating the CRN’s organizational characteristics, qualities and functions that influence the conduct of clinical research.	Articles evaluating aspects of the organizational activity of the CRN but unrelated to the conduct of clinical research.
**Comparator/context**	*Intentionally blank*	*Intentionally blank*
**Outcome**	Articles reporting research on the factors influencing CRN’s performance, impact and (enabling / barrier) characteristics in relation to conducting clinical research.	Articles reporting on measures unrelated to the CRN’s performance and impact in relation to conducting clinical research.
**Study characteristics**	Peer-reviewed original research	Articles that include reviews, editorials, letters, treatment consensus guidelines, conference addresses, abstracts and gray literature.
**Other**	Full-text articles that are available in English.	Any articles in languages other than English.

CRNs = Clinical Research Networks.

We utilized the Covidence systematic review software available via the University of Technology Sydney Library services to undertake and complete the review [10]. Covidence is a web-based collaboration software platform that streamlines the production of systematic and other literature reviews.

This project team includes experienced cancer doctors and trialists who are active contributors to cancer research and engaged in various CRNs around the world. Using a consensus from these subject matter experts, the protocol was informed by the Theory of Evidence-Based Medicine [11,12], including the search strategy, as well as the distillation of themes.

### Search strategy

Between 6 and 12 April 2023, we searched five electronic bibliographic databases (PubMed, Embase, Medline, CINAHL, Cochrane Central (which includes the Cochrane Database of Systematic Reviews to identify other related reviews) were systematically searched using a structured search strategy which included the following concepts (see Table 2 and Supplementary Materials Appendix A: Search Strategy). Consultation with a specialist university research librarian in January to April 2023 confirmed the search strategy, provided expertise and advice including the academic literature databases which were searched.

**Table 2.** Describes the key word search strategy and synonyms

Key word	Synonyms
Clinical research network (Title; Abstract) Search	clinical trial network research network consortium society (national adj3 institut*) (health adj3 institut*) (national adj3 collaborat*)
Clinical trial (Medical Subject Headings (MeSH); key word)	clinical trial “randomi?ed control* trial*”
Oncology (MeSH); key word)	oncology surgical oncology radiation oncology psycho-oncology

MeSH = Medical Subject Headings.

The search terms were adapted for use with other bibliographic databases in combination with database-specific filters. Aligned with the methods used by other highly-cited reviews [13] forward citation of the reference lists of the included articles were examined to identify additional relevant articles not found through database searches. Records identified via consultations with other expert and / or review of organizations’ websites were considered and hand-selected items were included in the analysis.

### Eligibility criteria

Articles were potentially eligible for inclusion if they reported research on factors influencing the performance of a cancer CRN in the conduct of its cancer clinical trials. A structured eligibility criteria framework in line with the PICOS framework [9] was applied to reviewing the full text articles (see Table 1). For a detailed description of the PICOS framework applied in this study see Supplementary Material Table 1.

### Selection of articles

Between May and October 2023, three reviewers (EJP, DD, TL) independently assessed for inclusion 10% of the initial title/abstract items to develop consistency of the process; discrepancies between the two data extractors were discussed, any disagreements were identified and resolved by discussion, and if agreement could not be reached the study was independently assessed by the third reviewer to achieve consensus. The remaining 90% of the initial title/abstract items were reviewed by one reviewer; less than 30 items were challenging to assess and these were circulated, considered and assessed by the three reviewers to reach a consensus.

### Data extraction and data entry

One reviewer from the research team extracted data independently using Endnote software and all available data were entered into the Covidence systematic review software. In the few cases where full text was not available efforts to request copies were communicated to the corresponding author and co-authors to request missing information via correspondence (email and phone), and data were extracted using the title/abstract review only. After uploading the articles from the search result, the automatic system in Covidence removed duplicate articles.

For each article that met the eligibility criteria and was included in this study, the following characteristics were analyzed against each aim:

1. *Identify and describe published original research which evaluates the performance of cancer CRNs that conduct cancer clinical trials, and characterize the organizational arrangements of each cancer CRN.*

A summary was prepared of the lead and collaborator cancer CRNs and other groups involved in each study. See Table 3.

**Table 3.** Description of the lead and collaborator cancer CRNs and collaborative groups involved in each of the selected studies

Article (year)	Name of lead cancer CRN/group	Name of collaborating cancer CRN/group
Monfardini [14]	International Society of Geriatric Oncology	NA
Denicoff [15]	American Society of Clinical Oncology	National Cancer Institute
Dandekar [16]	The Indian Council of Medical Research^	American Society of Clinical Oncology
Vose [17]	American Society of Clinical Oncology	Association of American Cancer Institutes^
Szczepanek [18]	American Society of Clinical Oncology	National Cancer Institute
Kim [19]	American Society of Clinical Oncology	Friends of Cancer Research^
Waterhouse [20]	American Society of Clinical Oncology	NA
Roth [21]	Children’s Oncology Group	Subcommittee: Adolescent and Young Adult Responsible Investigator Network
Rubio San Simon [22]	Spanish Society of Paediatric Haematology and Oncology	NA
Bagley [23]	Society of Neuro-Oncology	NA
Kizub [24]	African Organisation for Research and Training in Cancer	NA
Higgins [25]	National Cancer Institute National Clinical Trials Network	NRG Oncology Group^; The Alliance for Clinical Trials in Oncology^; Children’s Oncology Group; The Eastern Cooperative Oncology Group^#^; American College of Radiology Imaging Network^#^; The South Western Oncology Group.
Mittal [26]	Children’s Oncology Group	National Cancer Institute National Clinical Trial Network

NA = Not applicable.

^Organizations that did not meet the CRN definition were removed and excluded from analysis. See Supplementary Materials.

# Organizations that are recently established CRNs that formed by merging existing cancer CRNs. See Supplementary Materials.

Data for the selected cancer CRNs were collated to confirm the infrastructure and service provision arrangements for each CRN included in the articles in this analysis. Information from various public sources was collated including data accessed via their websites including corporate documents and other documentation and the National Institutes of Health National Library of Medicine website [27]. The characteristics of each cancer CRN were then collected: Name of organization, location (headquarter country, continent), membership-based regionality (national, global), membership discipline category (therapeutic area, oncology (subtype)), group maturity (calculated at 31 December 2023), incorporation status, membership structure (paid or fee-free), sources of financial support of each CRN), the CRN’s services to support research, access to policies to support research, the status of each CRN to act as a trial sponsor, the types of research activity (phases I to IV, other activities including registries) supported, the service provision arrangements supporting the IIT research capacity for each group (protocol development, data management, trial implementation, monitoring, analysis and reporting, results output support, annual meetings and member events) and the types of cancer clinical research developed by each CRN (phase I to IV, other types of research or no research). See Table 4.

**Table 4.** Characteristics of cancer CRN organization involved in each of the selected studies

Name of CRN [Acronym]	Location: Headquarter country, continent	Membership base	Group maturity [Year of establishment; Age^]	Incorporation status×	Membership terms >	Sources of financial support*	What services does the CRN provide to its members?^##^
International Society of Geriatric Oncology [SIOG]	Switzerland, Europe	Global	2000; 23	Inc.	Paid.	Govt., Phil., Comp., Comm.	Events.
American Society of Clinical Oncology [ASCO]	USA, Northern America	Global	1964; 49	Inc.	Paid.	Govt., Phil., Comp., Comm.	Prot., Data, Trial, Output, Events.
Children’s Oncology Group [COG]	USA, Northern America	Global	1955; 68	Inc.	Paid.	Govt., Phil., Comp., Comm.	Prot., Data, Trial, Output, Events.
Spanish Society of Paediatric Haematology and Oncology [SEHOP]	Spain, Europe	Spain	2007; 16	Unk.	Unk.	Govt., Phil., Uni.	Events.
Society of Neuro-Oncology [SNO]	USA, Northern America	Global	1994; 29	Inc.	Paid.	Govt., Phil., Comp., Comm.	Prot., Data, Trial, Output, Events.
African Organisation for Research and Training in Cancer [AORTIC]	Africa^#^	Global	1983; 40	Inc.	Paid.	Govt., Uni.	Prot., Data, Trial, Output, Events.
NRG Oncology Group † [NRG Oncology]	USA, Northern America	USA	2014; 9	Inc.	Paid+	Govt., Phil., Comp., Comm.	Prot., Data, Trial, Output, Events.
The Alliance for Clinical Trials in Oncology † [Alliance]	USA, Northern America	USA, Canada	2011; 12	Inc.	Paid++	Govt., Phil., Comp., Comm.	Prot., Data, Trial, Output, Events.
ECOG-ACRIN Cancer Research Group † {ECOG-ACRIN]	USA, Northern America	USA	2012; 11	Inc.	Paid.	Govt., Phil., Comp., Comm.	Prot., Data, Trial, Output, Events.
The South Western Oncology Group [SWOG]	USA, Northern America	USA	1956; 67	Inc.	Paid.	Govt., Phil., Comp., Comm.	Prot., Data, Trial, Output, Events.
National Cancer Institute [NCI]	USA, Northern America	USA	1937; 86	Oth. (Federal department)	Unk.	Govt.	Prot., Data, Trial, Output, Events.

CRN = Clinical Research Network.

^Age of the CRN operations are calculated from year of establishment to 31 December 2023.

×Incorporation status of each CRN, specify: Inc: Incorporated as a legal business entity, Uninc: Unincorporated (e.g., volunteer network), Oth: Other (Specify), Unk: Unknown.

# AORTIC operations are indicated as being based in Africa; however, the business registration is recorded as being situated in New York City, New York, USA. No reply correspondence to communications was received.

>Membership fees for each CRN, specify: Paid: Paid (with different categories), Free: Free or Unk: Unknown), + (subject to patient recruitment performance), ++ (subject to accrual, data quality and timeliness, adherence to policies and procedures, and participation in scientific activities).

* Sources of financial support to support the operations for each CRN, specify: Yes, specify: Govt: Government, Phil: Philanthropy, Comp: Competitive grants, Comm: Commercial partnership, Uni: University sources, N: No, Unk: Unknown.

†These CRNs recently formed as a result of a merging of pre-existing member-based CRNs (that now no longer exist). See Supplementary Materials.

## What services does the CRN provide to its members? Specify: Prot: Protocol development; Data: Data management; Trial: Trial implementation, monitoring, analysis and reporting; Output: Results output support, Events: Annual meetings and member events, and Oth: Other (specify).

To reflect contemporary activity only the cancer CRNs that are currently in operation were analyzed in this study and to minimize duplication cancer CRNs were only analyzed once in this study.

1. *Characterize the eligible articles included in this study*

The articles were evaluated and a description of each study which met the eligibility criteria was prepared, including: a description of the study, name of the responsible cancer CRN [Country], stakeholder participation (i.e., health consumers, industry representatives and / or multi-disciplinary teams of experts), number of responses (i.e., sample size) and number of cancer clinical trials evaluated in each study. See Table 5.

**Table 5.** Characteristics of the articles included in this review

Article (year)	Study description	Name of the responsible cancer CRN [Country]	Stakeholder consultation^	Number of responses (response rate%)	Number of cancer clinical trials evaluated
**Studies proposing factors influencing the success of CRNs and cancer clinical trials**					
Monfardini [14]	Results of a CRN member survey	International Society of Geriatric Oncology [Switzerland]	Patients Unk, Ind Unk, MDT Unk	58 (27%)	Not specified
Denicoff [15]	Recommendations from a CRN panel symposium, attendees were provided with relevant literature to review prior	American Society of Clinical Oncology [USA]	Patients Unk, Ind Unk, MDT Unk	358 (100%)	Not specified
Dandekar [16]	Results of a CRN member survey (distributed by email and in person at a conference)	American Society of Clinical Oncology^*^ [USA]	Patients Unk, Ind Unk, MDT Unk	587 (19%) [Email: 436/3,021; Conference: 151/151]	Not specified
Vose [17]	Results of an initial CRN member survey, followed by a CRN workshop attended by invited CRN members + stakeholders	American Society of Clinical Oncology [USA]	Patients, Ind, MDT	398 (32%) [Survey: 338/1,200; Workshop:>60]	Not specified
Szczepanek [18]	Results of a CRN member survey that was conducted pre- and post- attending a CRN symposium attended by invited CRN members and stakeholders	American Society of Clinical Oncology [USA]	Patients Unk, Ind Unk, MDT Unk	48 (100%)	Not specified
Kim [19]	Results of a CRN Working Group who held coordinated discussions with invited CRN members	American Society of Clinical Oncology [USA]	Patients Unk, Ind Unk, MDT Unk	Not specified	Not specified
Waterhouse [20]	Results of a survey to CRN members and stakeholders	American Society of Clinical Oncology [USA]	Patients Unk, Ind Unk, MDT Unk	32 (50%)	Not specified
Roth [21]	Results of a series of CRN webinars and workshops attended by invited CRN members	Childrens’ Oncology Group [USA]	Patients Unk, Ind Unk, MDT Unk	Not specified	Not specified
Rubio San Simon [22]	Results of a CRN member survey to evaluate clinical trial activity for the 2007 – 2020 period	Spanish Society of Paediatric Haematology and Oncology [Spain]	Patients Unk, Ind Unk, MDT Unk	Not specified	81 trials + 2 molecular platforms
Bagley [23]	Results of a CRN Think tank session, attended by invited CRN members and stakeholders, which made recommendation based on a data extraction and analysis of recruiting or not yet recruiting trials registered on the USA Clinical Trial Registry	Society of Neuro-Oncology [USA]	Patients, Ind, MDT.	Not specified	157
Kizub [24]	Results from a CRN Workshop and panel discussion attended by CRN members and stakeholders	African Organisation for Research and Training in Cancer [Africa^#^]	Patients Unk, Ind, MDT Unk	Not specified	Not specified
Higgins [25]	Results of a pilot study in which CRN members + stakeholders designed a principal investigator toolkit for enhancing accrual which was piloted in a CRN phase II/III trial	National Cancer Institute (NCI) National Clinical Trials Network [USA]	Patients, Ind Unk, MDT	Not specified	1 pilot trial
Mittal [26]	Results of a CRN member survey	Children’s Oncology Group [USA]	Patients Unk, Ind Unk, MDT Unk	58 (35%)	Not specified

CRN = Clinical Research Network.

^Description of which stakeholders were consulted and engaged in the study development and conduct activities, specify: Patients: Patients and or health consumer advocacy organizations; Patients Unk: Not specified in regard to health consumers; Ind: Industry (commercial) representatives and or organizations; Ind Unk: Not specified in regard to industry (commercial) representatives; MDT: Experienced experts from multi-disciplinary (MDT) clinical teams; MDT Unk: Not specified in regard to MDT clinical teams.

* Given that the Indian Council of Medical Research did not meet the cancer CRN definition, the lead cancer CRN responsible for Dandekar et al’s study is the American Society of Clinical Oncology (ASCO) [USA].

# The African Organisation for Research and Training in Cancer (AORTIC) operations are indicated as being based in Africa; however, the business registration is recorded as being situated in New York City, New York, USA. No reply correspondence to communications was received.

1. *Identify, define and characterize the factors influencing the CRN performance of the clinical trials they conduct*

We extracted data on the factors as defined by each article separately. Each factor, the subcategories for each factor, along with examples of the associated (barrier and enabling) characteristics were then evaluated in respect to the frequency that each factor appeared in each of the eligible articles. This information formed a ranking scale of frequency that each factor appeared in the eligible articles which represents the currently available original research published on these themes. To identify the comprehensiveness of each of the eligible articles, each of the eligible studies was then evaluated in respect to the number of the (newly-defined and newly-ranked) factors referenced in each study. See Table 6.

**Table 6.** Factors (ranked in respect to frequency), relevant subcategories and examples of the associated characteristics relevant to each study in this analysis

Rank	Factors	Sub-categories	Examples of characteristics	Article (year) in which the characteristic was identified
1	Site			Monfardini [14], Denicoff [15], Dandekar [16], Vose [17], Szczepanek [18], Kim [19], Waterhouse [20], Roth [21], Rubio San Simon [22], Bagley [23], Kizub [24], Higgins [25], Mittal [26].
		Team	Appropriate roles and responsibilities. Access to available experts (with consideration to degree of previous clinical research experience, track-record), existing referral pathways between specialists. Access to suitable clinics, tumor boards, MDTs and case conferences. Identification of a trial champion. Degree of communication between site staff. Degree of variability of research activity within departments and hospitals. Degree of culture and leadership influence (considerations to senior leadership engagement, institutional commitment, MBA-style leadership assessment on clinical leaders, including a shared vision and purpose).	
		Protocol development and design	Site feasibility and evaluation (e.g., application of site benchmarks (expected vs actual accrual)). Degree of contribution to recruitment planning estimates. Degree of input into protocol design elements; Degree of time / effort invested with sponsors modifying protocols. Degree of pragmatism selecting studies.	
		Resourcing and Incentivization	Degree of funding provided (i.e., site payments), development of financial models (including site, Medicare, insurance), degree of time allocated and allocation of resourcing (staff FTE to support research workload +- adequate and / or protected time), number and complexity of trials, mechanism for research prioritization (and increasing resourcing to support doctors to prioritize their time). Degree of encouragement and “buy in.” Clearly defined value proposition to justify involvement to support trials.	
		Trial access	Simplification of administrative burdens and centralized processes. Development of templates, centralized processes (i.e., institutional processes and best practice) and revisiting modeling during the study design phase, as well as during study conduct. Degree of logistical constraints of accessing studies (e.g., institutional challenges between smaller and tertiary centers, ethics and governance, Medicare, insurance challenges). Degree of competition (nationally, globally).	
		Trial infrastructure	Need for centralization of administrative processes including standard SOPs, master templates (including standardized costs and financial information), agreements, financial models, suitable IT systems (e.g., databases to more accurately assess feasibility contributions, patient medical records, databases to maintain patient follow up, financial systems). Need for physical space.	
		Education and training	Adequately knowledgeable staff, quality of training and CPD accreditation processes, ICH-GCP training, general clinical trial training (including access to resources), protocol-specific clinical trial training, management systems. Use of Toolkits (e.g., PowerPoint slides, websites, brochure, email template, video, video script, presentation session, social media updates (e.g., X formerly known as Twitter, Facebook).	
		Marketing and communications	Site staff engagement and communication initiatives and resources (e.g., personal connection beyond emails (via phone and in person), newsletters, use of social media, trial promotion materials (e.g., toolkits, flyers, websites), study-specific training materials, websites, videos, use of registries, health literacy tools, simplified site consent forms, use of decision aids). Development of trial progress reports. Opportunities for research output and/or speaking opportunities at conferences and forums.	
		Data management and audit	Suitable IT systems at site to support trial activity, study screening logs, data management, patient follow up, and auditing functions. Ability to support sponsor visits (initiation, monitoring and close out). Ability to manage documents (including regulatory, safety events (i.e., Adverse events and Serious Adverse Events), and resolution of data queries.	
		Governance	Clear identification of stakeholders. Appropriate contracting, templates, negotiation pathways to minimize bureaucracy, including medical insurance reimbursements and other multi-stakeholder issues related to clinical trial conduct. Access to legal expertise.	
2	CRN			Monfardini [14], Denicoff [15], Dandekar [16], Vose [17], Kim [19], Waterhouse [20], Roth [21], Rubio San Simon [22], Bagley [23], Kizub [24], Higgins [25], Mittal [26].
		Team	Identification and appropriate roles and responsibilities across stakeholder matrix. Degree of intergroup collaboration and partnership. Creation of trial champions. Degree of job satisfaction and staff retention. Culture, shared vision and purpose. Awareness of possible crossover between CRN members and Site staff.	
		Protocol development and design	Innovation (quality ideas, quality trials for under-research diseases with high-patient need (e.g., rare diseases). Considerations including sample size calculations, study design, endpoints, eligibility criteria, performance status, study visit scheduling, monitoring frequency, safety and quality assurance metrics. Use of e-consent, telehealth, remote monitoring, remote lab results, remote patient (and safety) monitoring, remote investigational product supply, streamlined data collection (in alignment with standard of care). Research prioritization and consideration of real-world setting. Considerations of analysis to maximize data utility, research findings and application. Willingness to amend protocols.	
		Resourcing and Incentivization	Degree, availability and allocation of resources, time, money, site payments, staffing. Research prioritization. Reward and recognition for effort and performance (e.g., site capitation, research team opportunities to present at conferences / forums, degree of “buy in”). Clearly defined value proposition to justify involvement to support trials.	
		Trial access	Site location and speciality, track-record, previous clinical trial experience, degree of demonstrated site performance and achievement, key opinion leader considerations. Degree of Competition (nationally, globally). Willingness to add new sites during trial lifecycle.	
		Trial infrastructure	Processes, e.g., ethics, regulations, safety, IT and project management systems to support CRN and members.	
		Education and training	General clinical trial and protocol-specific training materials, training tools, templates, screening logs, sponsor-led training sessions. Monthly webinars (with sites). Annual CRN meetings and forums.	
		Marketing and communications	Stakeholder engagement and communication initiatives (e.g., personal connection beyond emails (via phone and in person), hosting of annual meeting, use of clinical registries, use of social media, development of trial newsletters, trial awareness initiatives (e.g., websites, videos, registries, health literacy tools, simplified consent forms, decision aids), research output speaking opportunities (at conferences and forums), regular circulation of templates (e.g., trial progress reports), growing commercial marketing approaches).	
		Data management and audit	Sufficient technology solutions to support efficient trial conduct. Adequate instructions, policies and procedures. Appropriate training and expectations around timelines with sites.	
		Governance	Clear identification of stakeholders. Appropriate contracting (e.g., concerning funding, IP, confidentiality, degree of transparency for inter-institutional protocol assessments, Sites, CROs, third party agreements). Development of templates, negotiation pathways to minimize bureaucracy, including medical insurance reimbursements and other multi-stakeholder issues related to clinical trial conduct (e.g., contracts and compliance). Appropriate structures to monitor trial progress (meetings for sponsor +- site, review of screening logs, oversight committee, project management committees and meetings arrangements).	
3	Patient			Monfardini [14], Denicoff [15], Szczepanek [18], Kim [19], Waterhouse [20], Bagley [23], Kizub [24], Higgins [25].
		Patient considerations	E.g., Co-morbidities, age, carer influences, health literary levels, financial circumstance, support systems, incentive (e.g., patient’s willingness to participate)	
		Trial access	Sites proximal to patients and their families. Access to telehealth systems. Referral bias. Access to health literacy tools, simplified consent forms, decision aids. Early withdrawal or failure to complete follow up.	
		Degree of health consumer representation	Integrated health consumer review mechanisms. Nomination of health consumer representative on research committees / panels. Health consumer input on progress reporting.	
4	Regulatory			Vose [17], Kim [19], Waterhouse [20], Roth [21], Rubio San Simon [22], Bagley [23], Kizub [24].
		Compliance with administration, policy and procedures	Clinical trial regulatory approval processes via centralized national systems. Mandatory Sponsor-specific requests (e.g., certification, approval). Federal mandates and policy recommendations (e.g., policy promoting intergroup collaboration, partnership and research priorities).	
		Harmonization and centralization of accreditation of systems	Establish electronic health record nationally. Standardize guidelines, training, trial protocols and processes (site- and national level). Standardize and implement Good Clinical Practice training systems and processes (nationally) (e.g., guidelines, training, trial processes)	
		Drug approval and marketing approval mechanisms	Regulatory-approved processes re trials, drug approvals and indications. Regulatory-approved conventions (with consideration to existing requirements and new options, e.g., adaptive platform trial designs).	
		Human Research Ethics Committees	Application (submission and approval) processes and timelines. Guidance with regard to existing-approved conventions (with consideration to inclusive trial design and flexible eligibility criteria).	
		Medicare and health insurers	Coverage and mapping analytics and guidance on Medicare and insurance reimbursement mechanisms (and their application in the context of the USA operations).	
5	Policy		Monfardini [14], Denicoff [15], Szczepanek [18], Bagley [23].	
		Availability to published evidence-based information	Best practice recommendations, national treatment guidelines, institutional policies and standard definitions to manage patients.	
6	Industry		Rubio San Simon [22], Kizub [24].	
		Degree of engagement and support	Access to industry opportunities (e.g., for trial consideration, commercial marketing approaches, speaking opportunities, collaboration). Access to CRO services. Degree of investment and resourcing availed.	

CRN = Clinical Research Network; MDT = Multi-Disciplinary Teams; MBA: Master of Business Administration; CPD = Continuing Professional Development; ICH-GCP = International Council for Harmonisation’s Good Clinical Practice (GCP) guidelines, an international standard for ethical and scientific quality in clinical trials involving humans; GCP = Good Clinical Practice; FTE = Full time equivalent; IRB = Institutional Review Board; HREC = Human Research Ethics Committee; SOPs = Standard Operating Procedures; IT = Information Technology; AEs = Adverse Events; SAEs = Serious Adverse Events; IP = Intellectual Property; CRO = Clinical Research Organization.

1. *With consideration to the types of evidence available and analyzed in this study, identify any knowledge gaps to allow for the identification of future research initiatives.*

### Assessment of bias/quality

Reviews were quality rated by a single reviewer. No formal methods were used to examine bias across the articles as the designs of each study were too diverse and not all lending themselves to appraisal with checklists.

### Analysis and synthesis

We took a narrative approach to the synthesis of the data [28]. The definition of the research question and identification of themes of interest forming the study protocol (including the eligibility criteria, alongside the development and implementation of the PRISMA statement [29] and PICOS framework [30] to enhance the specificity of the factors, subcategories for each factor were identified and defined. A summary of the factors that were identified and relevant subcategories (including examples of the (barrier and enabling) characteristics) was then prepared, this information was evaluated in respect to relevance for each of the factors (and relevant subcategories).

Analysis, translating and synthesis of the data were conducted collaboratively across multiple meetings and communications throughout 2023–2024.

## Results

**Figure 1.**
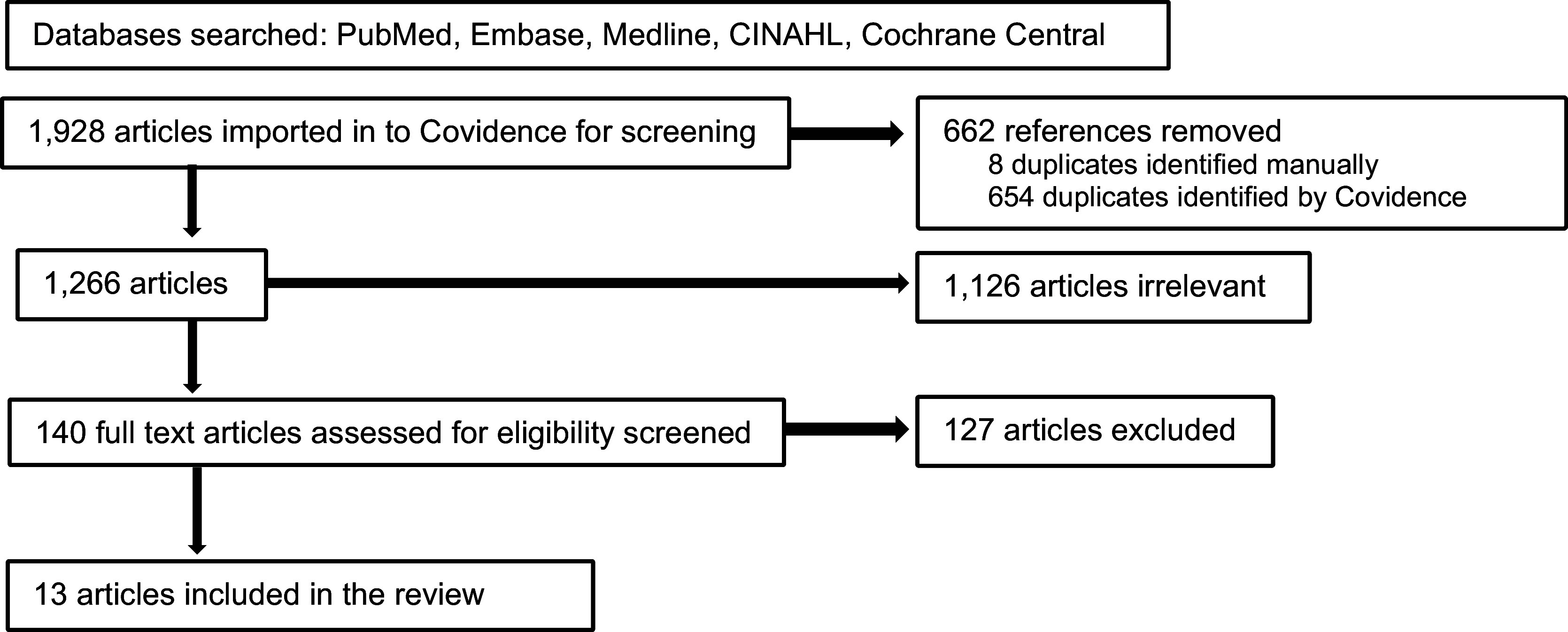
Summary flow diagram for the systematic review.

The database searches identified 1,928 articles, of these 662 were duplicates, leaving 1,266 articles which were screened as title/abstract format. Of these, 140 full text articles were reviewed and 13 articles [14–26] were accepted for inclusion. A summary flow diagram describes the screening process (see Figure 1). The full PRISMA Flow Diagram is available, see Supplementary Materials: Figure 1.

For the screened but ineligible articles identified in this study: Various cancer CRNs operating worldwide have published on similar themes however many articles were ineligible for inclusion. Several selected articles included older CRNs that no longer exist (and have more recently merged into a newer entity) (see Supplementary Materials: Table 2), several entities failed to meet the definition of being a CRN (see Supplementary Materials: Table 3), a further 25 cancer CRNs were identified in ineligible articles (see Supplementary Materials: Table 4) and we identified ineligible contributions from cancer CRNs researchers in 12 countries and 3 continents (self-described in each of their articles) (see Supplementary Materials: Table 5). All screened but ineligible articles excluded from the review are available (see Supplementary Materials: Table 6).

### Key study features

- Thirteen articles published between 2007–2022 were included in the review,
- Eleven cancer CRNs were identified (including eight in the USA, one each in Spain, Switzerland and Africa),
- This study broadly defined six factors (Site, CRN, Patient, Regulatory, Policy and Industry, supported by subcategories for each factor) that influenced the performance of these organizations to complete their clinical trials.
- The articles from each organizations reflect mature research operations (the youngest had been operating for nine years, and the oldest had been operating for 87 years), of these nine organizations offered IIT trial development services to their members, of these eight were able to technically offer legal trial sponsorship services,
- Only three articles relied on any actual cancer clinical trial data to evaluate performance, and
- No best-practice frameworks to enhance trial success were identified.

In structuring the presentation of the remainder of the results section, the findings have been mapped to the research questions.

1. *Identify and describe published original research which evaluates the performance of cancer CRNs that conduct cancer clinical trials, and characterize the organizational arrangements of each cancer CRN.*

From the 13 articles identified in this study, 11 cancer CRNs were identified, and the lead and collaborator groups involved in each study are summarized in Table 3.

Table 4 describes the characteristics of the 11 cancer CRNs identified in the articles included in our review. Despite the best efforts to obtain all of the information, in some cases some organizational data was unable to be collected. For the 11 cancer CRNs identified in our analysis:

Eight organizations are based in North America (all eight are headquartered in the USA), two in Europe (one headquartered in Switzerland, one headquartered in Spain) and one in Africa.

Six organizations were designed to support a strictly national membership base; the remaining five were designed to attract, retain and support a global membership base.

The maturity of the 11 organizations ranged from nine years to 86 years of research operations, averaging 37 years of operations since establishment (calculated to 31 December 2023). The most recent group having been established in 2014; the most mature group having been established in 1937.

Nine organizations were formally incorporated as legal business entities. Despite the best efforts, data for one entity was unable to be obtained (i.e., SEHOP) and one entity operates as a federal government department reporting to USA Congress and is solely reliant on government support (i.e., NCI).

Whilst all 11 organizations in this study indicated that they received government financial support, nine offer structured membership application arrangements with an annual fee payable to the organization. Of these, only two offered membership terms subject to specific performance targets (e.g., demonstrable patient recruitment, data quality, timeliness, adherence to policies and procedures, member participation in CRN annual meetings) (i.e., NRG Oncology and Alliance). These data were not able to be obtained for two groups (i.e., SEHOP and NCI). The remaining ten organizations receive multiple sources of financial support (including a mix of various sources including government, philanthropy, competitive grants, commercial partnerships) and of these, only two specifically identified university sources of financial support).

Of the 11 organizations identified in this study, three entities were relatively recently established (i.e., NRG Oncology, Alliance and ECOG-ACRIN), emerging from USA membership-based CRNs that now no longer exist. See Supplementary Material: Table 3.

The service provision arrangements supporting IIT research for each of the 11 organizations was then evaluated. Nine entities offered a variety of operational services and service provision arrangements supporting IIT research capacity to their membership base, including at a minimum: protocol development, data management, trial implementation, monitoring, analysis and reporting, administrative support for research results and associated output (e.g., opportunities for posters, publications, presentations) and CRN-hosted annual meetings and member events. The remaining two entities offered and conducted CRN-hosted annual meetings and / or member events to their membership. Only one entity had no public facing information regarding minimum policies and procedures via their website, the remaining ten entities granted general access to this operational information via their websites. Eight organizations were able to function as the legal trial sponsor for phase I–IV IIT clinical research activity. For the three entities who were not set up to act as the trial sponsor (i.e., SIOG, SEHOP and AORTIC) they did not offer protocol development services to their members. The characteristics of cancer CRN organization involved in each of the selected studies; including the service provision arrangements supporting the phase I–IV IIT clinical research capacity are described in Supplementary Materials Table 7.

1. *Characterize the eligible articles included in this study*

Table 5 describes the 13 selected articles that met the eligibility criteria. All 13 articles were published between 2007–2022; 12 in the past decade, of these six were published since the COVID-19 pandemic was declared in early 2020. Three studies analyzed trial data (i.e., two studies utilized mixed methods (one generated and pilot evaluated a site toolkit with the suggestion of it being utilized in future research to evaluate its utility to optimize trial performance) and one study utilized quantitative methods (performing an analysis of NIH CT.gov derived metrics [27]) and ten studies recommended best-practice based on expert opinion via qualitative methods (e.g., surveys). Scholars who support the Theory of Evidence-Based Medicine recognize expert opinion as being lower quality evidence to guide practice [11,12].

For the 13 eligible articles, four cancer CRNs (based and headquartered in North America) lead ten of the eligible studies (i.e., ASCO lead six studies, COG lead two studies, SNO and NCI each lead one individual study), two cancer CRNs based and headquartered in Europe each lead one individual study (i.e., SIOG and SEHOP) and one cancer CRN based and headquartered in Africa lead one study (i.e., AORTIC). Seven articles provided information on the number of responses (ranging from 32 to 587 reflecting a response rate 27% to 100%); average 219 responses per study (calculated only in respect to those studies that included this metric in their manuscript); the remaining six studies did not include any reference to the sample size or number of responses used to inform their research and study publications.

Three articles specified who and which stakeholders were engaged and involved in their study design and reporting. Higgins [25] developed site tool kit and undertook a pilot evaluation with comprehensive input from health consumers and MDT experts. Vose [17] and Bagley [23] described comprehensive stakeholder engagement in the development of their study design and reporting (engaging health consumers, industry and MDT experts and representatives). Kizub [24] described comprehensive stakeholder engagement in the development of their study design and specified the involvement of industry representatives in their acknowledgment section (specifically for the financial support provided by industry to support the study activities outlined in the manuscript).

Ten studies did not specifically specify the number of clinical trials or types of research study that were being evaluated. Of the three studies that provided this information, one study evaluated the results drawing on data related to 81 trials and two molecular platforms [22], one study evaluated the results drawing on data related to 157 trials [23] and one study for a single pilot project for a single trial [25].

No study indicated human research ethics committee approval was obtained for any of the 13 studies. Ten studies omitted reference to ethics arrangements altogether, three studies specified that ethics considerations were not necessary and were therefore not obtained.

1. *Identify, define and characterize the factors influencing the CRN performance of the clinical trials they conduct*

All 13 articles reported various influences associated with the performance of their cancer CRN in its conduct of their cancer clinical trials. The influences were identified in each article as the associated characteristics describing either enabling or barrier characteristics related to the CRN performance and its clinical trial activity. These were then evaluated, considered and categorized into grouped themes and these defining (enabling or barrier) characteristics forming the factors. The characteristics broadly fell in to six factors: Site, CRN, Patient, Regulatory, Policy and Industry; with associated subcategories defined for each factor. The factors and the respective subcategories can apply interchangeably to classify as being either an enabling or barrier characteristics depending on circumstance. We analyzed each article (see Supplementary Materials: Table 8). The descriptions of the six factors were then pooled and these were ranked in respect to the frequency (i.e., whether the factor was identified in each study or not); these results are summarized in Table 6, below.

In our analysis, the factors (ranked in order of the frequency) identified as being an influencing factor on the performance of cancer CRNs to ensure clinical trial success included:

- All studies identified site factors;
- 12/13 studies identified the CRN factors;
- 8/13 studies identified patient factors;
- 7/13 studies identified regulatory factors;
- 4/13 studies identified the role of policy factors; and
- 2/13 studies recognized role of industry factors.

The average number of factors included in each article was three, ranging from two to five, and no study addressed all six factors. Bagley [23] and Kizub [24] were the most comprehensive with each study including five factors; including site, CRN, patient, regulatory, the same with the exception for the influence of policy [24] and industry [23].

1. *With consideration to the evidence currently available, identify any knowledge gaps to allow for the identification of future research initiatives.*

The selected studies were relatively current (2007–2022) using mainly qualitative methods (e.g., the results of surveys, symposiums, workshops, panel, webinars, seminars, think-tank activities) and the evaluations were broad in scope relating to general oncology themes and experiences conducting any clinical research activities reflect a North American perspective. There was only one study that evaluated the results of an intervention within a cancer clinical trial setting [25].

## Discussion

This review has identified a limited body of original research drawing mainly on expert opinion primarily offering a North American perspective engaging with the USA health system [15, 17–21, 23, 25, 26]. This study has identified and defined six factors that researchers report as being influential on the performance of cancer CRNs to ensure clinical trial success (i.e., site, CRN, patient, regulatory, policy and industry factors) and that these factors are common across the studies (and the countries / continents / members represented by each cancer CRN) in our review.

Despite the huge volume of activity and investment in global cancer clinical research [31,32], we have not identified a best-practice framework for trial sponsors to refer to when commencing the development and implementation of new cancer clinical research to maximize study success. Whilst we acknowledge that there is an existing body of literature outside of the CRN setting to improve accrual and maximize trial availability [33,34], our study suggests that there are under-researched areas including the CRN sector and those factors less frequently reported (see Table 6). Indeed, maybe there are other factors that have not yet been identified or studied or perhaps some may not warrant influence (e.g., industry factors) as these are highly-dependent on the cancer CRN speciality.

Clinical trials operate in a tightly regulated environment [35–38] and are complicated to establish and complete. Combining these realities with the lengthy grant applications processes necessary to initiate IIT clinical research, this makes the completion of IIT clinical research in the cancer CRN sector complex. This study has identified and mapped a global network of cancer CRNs (see Tables 3, 4; Supplementary Materials: Tables 1, 2, 3, 4 and 5). Each of these organizations reflect their local governance arrangements in respect to membership engagement, regulatory, ethics and site requirements, reimbursement and insurance mechanisms, patient and health consumer engagement, as well as infrastructure arrangements sustaining their operations. We acknowledge that there are a number of cancer CRNs which were excluded from our study (as they did not meet the inclusion criteria) who have published work on similar themes which may be of interest (e.g., including but not limited to citations [39–45].

Despite the variation in oncology treatments across different stages of disease (early to late) and multi-modality treatments, in our study there was an absence of published research specifying, defining or analyzing the different requirements of different types of clinical research (earlier to later phase trials) for the same disease. The articles in this study focus mainly on general oncology clinical research experiences (with the exception of geriatric oncology via SIOG, pediatric oncology via COG and SEHOP, neuro-oncology subspecialties via SNO) and the studies all coded clinical trial research to include any study design (earlier to later phase) as being the equivalent parameter. Only three studies specifically mentioned the relevance of multi-disciplinary team (MDT) expertise in treating cancer patients, as well as the role of health consumer advocacy contributions and how both MDTs and patients / health consumers informed study designs, data collection and reporting. In our study, there was limited acknowledgment of role and impact of experienced trialists, principal investigators (responsible for new idea generation, trial design and implementation, reporting and delivering the best treatments to patients) and the patient/health consumer voice (in their role advocating for optimal outcomes for healthcare users, carers and the community more broadly through the conduct of high-quality clinical research).

### Impact of COVID

Whilst six of the identified studies were published during the COVID-19 pandemic, only two studies referred to the impact of COVID-19 pandemic in transforming the global clinical trial landscape [20,25]. Both of these studies identify the role of the pandemic in influencing the broader clinical research community to embrace new initiatives that improve efficiencies whilst maintaining safety and ethical standards (e.g. swifter regulatory and ethical processes for trial registration, trial monitoring and drug approvals, prioritized research questions that prioritize particular patient outcomes and endpoints, streamlined study designs, telehealth services (enabling closer to home care +- remote patient monitoring), more nimble sponsor processes including remote sponsor visits (initiation, monitoring and close out) and centralized data monitoring (including pragmatic safety reporting requirements). These studies identified that “*many of the adaptations to trials made during the pandemic provide a long-term opportunity to improve and transform the clinical trial system*” (page 417, [20]).

An example of an Australian initiative providing long-term transformation to the clinical trial system was the establishment of national telehealth initiatives. Driven by the need to improve health care access nationally across the continent for the one third of all Australians living and working outside of the metropolitan centers, Dr Sabe Sabesan, commenced investigation of the benefits of telehealth initiatives well in advance of COVID-19 pandemic [46–53]. With the backing of the Clinical Oncological Society of Australia (COSA) circa 2017, this initiative secured national support enabling more patients access to more trials (closer to home) and primed the Australian clinical trial community for the challenges presented by the pandemic highlighting “*the significant advantages and utility of the TeleTrial Model in Australia and internationally*” (page 5, Section 4.1, COSA press release 2021) [54].

There were only two other screened studies that reported an impact of COVID-19 pandemic but both were excluded as they did not meet the PROSPERO protocol inclusion criteria for original research methods. Pothuri et al. summarized revised clinical practice guidelines for the gynecology oncology network in response to the pandemic [55] and the South Western Oncology Group group demonstrated a reduction in recruitment to a radiotherapy trial [56]. Another recent study produced by the Spanish Society of Paediatric Haematology and Oncology group [28] reported on their experiences during COVID-19 however this study was not identified in this review [57].

### Future research directions

Cancer CRNs play a vital role in medical research globally. Research and new knowledge generation may help to maximize clinical trial success and this ultimately leads to better patient outcomes and impact. Designing and monitoring easily accessible data to guide cancer CRNs to improve trial success, has the potential to inform more efficient use of limited resources and so generate evidence more swiftly to improve patient outcomes. This is an emerging area of research. Of particular interest are Australian authored and Australian cancer CRN contributions; in both cases this study demonstrates that there appears to be limited available original research publications and future research is warranted.

Given the depth of experience of many CRNs, some groups may already be in a position to quantify and measure (potentially already known) metrics and this may help to improve performance. Easily measurable metrics that could be assessed include: strategies to support CRN members and participating sites (e.g., previous track-record recruiting to trials (initiated, completed, closing, published), intervention type, funding and site/patient payments, training and education initiatives, access to tumor-relevant MDT, development of trial-specific research output (videos, presentations, publications), site and patient payments), strategies to support the CRN (e.g., funding, pilot grants, CRN staff training); or strategies to engage patients / health consumers (e.g., input into protocol design, site accessibility considerations, patient-facing study promotion materials).

Additionally, research evaluating CRNs located and operating outside of North America may allow for exploration of fresh perspectives. This may add depth to what is already known. For example: Regions outside of North America experience differences in the regulatory landscape, policy frameworks, public versus private healthcare arrangements. These contexts may influence initiation, conduct and completion of important clinical research. Future research on these themes may provide a more rounded perspective on the global influence and impact of the work produced by cancer CRNs with the support of their members.

## Limitations

Due to the heterogeneity of the articles, several decisions were made during the selection criteria process which potentially skewed or limited results. We limited the search strategy to refer to original research articles for three search terms (“Clinical research networks,” “Clinical trials” and “Oncology” (and associated synonyms for each term)) to restrict the search to IIT research activity. While the decision to exclude studies which referred to clinical research more broadly (regardless of sponsor-type) may have excluded relevant articles, our strategy led to including only the most relevant articles to answer our question. Exclusion of papers published in languages other than excluded potential research on cancer CRN activity from non-English speaking parts of the world.

We acknowledge the small sample involving only a small number (13) of eligible articles representing the activities of only 11 cancer CRNs worldwide (involving 2,464 subjects each drawing on their own clinical research experiences, including specific reference to 241 clinical research activities in three articles). A systematic quality analysis was not performed for any of the articles as the designs were too diverse and not all lending themselves to appraisal with available checklists. We acknowledge any perceived or potential publication bias that may arise.

While the findings of this study have the potential to inform the development of a framework resource, such an undertaking falls outside the scope of the current review. Future research is being considered by members of this research team.

## Conclusion

For the first time, this study has reviewed the current literature and in doing so identified 11 membership-based cancer CRNs, predominately representing a north American perspective, who have each published on their factors influencing their performance generating, conducting and completing their clinical research. In this review, we have not identified a best-practice framework but have identified and defined six factors (i.e., site, CRN, patient, regulatory, policy and industry, supported by subcategories for each factor), that influence the performance of these organizations to successfully accrue to and complete their clinical trials.

The data from this study proves that more research, including from other cancer groups and regions in the world, would add depth to what is already known. By defining and describing common factors, alongside easily measurable characteristic metrics, the process of systematically mapping influences that affect organizations who conduct and run IITs has commenced. Future research may build on these findings to inform the development of a best-practice framework to help guide existing and newly forming CRNs, and possibly other research organizations who sponsor trials, to optimize trial success. More research building on these findings merits further evaluation.

## Supporting information

10.1017/cts.2025.10210.sm001 Paton et al. supplementary material Paton et al. supplementary material

## Supplementary material

The supplementary material for this article can be found at https://doi.org/10.1017/cts.2025.10210.

## References

[1] Institute of Medicine (US) Committee on Cancer Clinical Trials and the NCI Cooperative Group Program. A National Cancer Clinical Trials System for the 21st Century: Reinvigorating the NCI Cooperative Group Program. Nass SJ , Moses HL , Mendelsohn J , editors. Washington, DC: National Academies Press (US), 2010. 25032387

[2] Murthy VH , Krumholz HM , Gross CP. Participation in cancer clinical trials: Race-, sex-, and age-based disparities. JAMA. 2004;291:2720–2726. doi: 10.1001/jama.291.22.2720. 15187053

[3] Tejeda HA , Green SB , Trimble EL , et al. Representation of African-Americans, Hispanics, and whites in National Cancer Institute Cancer Treatment Trials. J Natl Cancer Inst. 1996;88:812–816. doi: 10.1093/jnci/88.12.812. 8637047

[4] Unger JM , Cook E , Tai E , Bleyer A. The role of clinical trial participation in cancer research: Barriers, evidence, and strategies. Am Soc Clin Oncol Educ Book. 2016;35:185–198. doi: 10.1200/EDBK_156686. 27249699 PMC5495113

[5] Stensland KD , Kaffenberger SD , George AK , et al. Prostate cancer clinical trial completion: the role of geography. Contemp Clin Trials. 2021;111:106600. doi: 10.1016/j.cct.2021.106600. 34673273 PMC8908357

[6] Tran G , Harker M , Chiswell K , et al. Feasibility of cancer clinical enrolment goals based on cancer incidence. JCO Clinical Cancer Informatics. 2020;4:35–49. doi: 10.1200/CCI.19.00088. 31977253

[7] Arksey H , OMalley L. Scoping studies: Towards a methodological framework. Int J Soc Res Method. 2005;8:19–32. doi: 10.1080/1364557032000119616.

[8] Levac D , Colquhoun H , O’Brien KK. Scoping studies: Advancing the methodology. Implementation Sci. 2010;5:69. doi: 10.1186/1748-5908-5-69. PMC2954944 20854677

[9] Richardson WS , Wilson MC , Nishikawa J , Hayward RS. The well-built clinical question: A key to evidence-based decisions. ACP J Club. 1995;123:A12–A13. 10.7326/ACPJC-1995-123-3-A12 7582737

[10] Covidence systematic review software, Veritas Health Innovation, Melbourne, Australia, (https://www.covidence.org) Accessed January 2023 – January 2024.

[11] Rogers W , Hutchison K. Evidence based medicine in theory and practice: epistemological and normative issues. In: Schramme T , and Edwards S. eds. Handbook of the Philosophy of Medicine. Dordrecht: Springer, 2017: 851–872. Part V Medical Knowledge, Chapter 52. ISBN: 978-94-017-8687-4.

[12] Oxford Centre for Evidence Based Medicine, Levels of Evidence, (https://www.cebm.ox.ac.uk/resources/levels-of-evidence/ocebm-levels-of-evidence) Accessed January 2024.

[13] Debono DS , Greenfield D , Travaglia JF , et al. Nurses’ workarounds in acute healthcare settings: A scoping review. BMC Health Serv Res. 2013;13:175. doi: 10.1186/1472-6963-13-175. 23663305 PMC3663687

[14] Monfardini S , Aapro MS , Bennett JM , et al. Organization of the clinical activity of geriatric oncology: Report of a SIOG (International Society of Geriatric Oncology) task force. Crit Rev Oncol Hematol. 2007;62:62–73. doi: 10.1016/j.critrevonc.2006.10.003. 17300950

[15] Denicoff AM , McCaskill-Stevens W , Grubbs SS , et al. The National Cancer Institute-American Society of Clinical Oncology Cancer trial accrual symposium: Summary and recommendations. J Oncol Pract. 2013;9:267–276. doi: 10.1200/JOP.2013.001119. 24130252 PMC3825288

[16] Dandekar M , Trivedi R , Irawati N , et al. Barriers in conducting clinical trials in oncology in the developing world: A cross-sectional survey of oncologists. Indian J Cancer. 2016;53:174–177. doi: 10.4103/0019-509X.180865. 27146772

[17] Vose JM , Levit LA , Hurley P , et al. Addressing administrative and regulatory burden in cancer clinical trials: Summary of a stakeholder survey and workshop hosted by the American Society of Clinical Oncology and the Association of American Cancer Institutes. J Clin Oncol. 2016;34:3796–3802. 27601549 10.1200/JCO.2016.69.6781

[18] Szczepanek CM , Hurley P , Good MJ , et al. Feasibility of a centralized clinical trials coverage analysis: A joint initiative of the American Society of Clinical Oncology and the National Cancer Institute. J Oncol Pract. 2017;13:395–400. doi: 10.1200/JOP.2016.020313. 28481681

[19] Kim ES , Bruinooge SS , Roberts S , et al. Broadening eligibility criteria to make clinical trials more representative: American Society of Clinical Oncology and friends of cancer research joint research statement. J Clin Oncol. 2017;35:3737–3744. doi: 10.1200/JCO.2017.73.7916. 28968170 PMC5692724

[20] Waterhouse DM , Harvey RD , Hurley P , et al. Early impact of COVID-19 on the conduct of oncology clinical trials and long-term opportunities for transformation: Findings from an American Society of Clinical Oncology Survey. JCO Oncol Pract. 2020;16:417–421. doi: 10.1200/OP.20.00275. 32396491

[21] Roth M , Mittal N , Saha A , Freyer DR. The children’s oncology group adolescent and young adult responsible investigator network: A new model for addressing site-level factors impacting clinical trial enrollment. J Adolesc Young Adult Oncol. 2020;9:522–527. doi: 10.1089/jayao.2019.0139. 32077782 PMC7415882

[22] Rubio-San-Simón A , Hladun AR , Juan RA , et al. The paediatric cancer clinical research landscape in Spain: A 13-year multicentre experience of the new agents group of the Spanish Society of Paediatric Haematology and Oncology (SEHOP). Clin Transl Oncol. 2021;23:2489–2496. doi: 10.1007/s12094-021-02649-y. 34076861

[23] Bagley SJ , Kothari S , Rahman R , et al. Glioblastoma clinical trials: Current landscape and opportunities for improvement. Clin Cancer Res. 2022;28:594–602. doi: 10.1158/1078-0432.CCR-21-2750. 34561269 PMC9044253

[24] Kizub D , Manner CK , Graef K , et al. Action for increasing diversity, market access, and capacity in oncology registration trials-is Africa the answer? Report from a satellite session of the accelerating anti-cancer agent development and validation workshop. JCO Glob Oncol. 2022;8:e2200117. doi: 10.1200/GO.22.00117. 35714309 PMC9232363

[25] Higgins KA , Thomas A , Soto N , et al. Creating and implementing a principal investigator tool kit for enhancing accrual to late phase clinical trials: Development and Usability study. JMIR Cancer. 2022;8:e38514. doi: 10.2196/38514PMID:36006678. 36006678 PMC9459930

[26] Mittal N , Langevin AM , Kyono W , et al. Barriers to pediatric oncologist enrollment of adolescents and young adults on a cross-network national clinical trials network supportive care cancer clinical trial. J Adolesc Young Adult Oncol. 2022;11:117–121. doi: 10.1089/jayao.2021.0041. 33983848 PMC8864435

[27] National Institutes of Health, National Library of Medicine Clinical Trials Registry website, (https://clinicaltrials.gov/) Accessed: January 2023 – January 2024.

[28] Rodgers M , Sowden A , Petticrew M , et al. Testing methodological guidance on the conduct of narrative synthesis in systematic reviews: Effectiveness of interventions to promote smoke alarm ownership and function. Evaluation. 2009;15:49–73.

[29] Moher D , Liberati A , Tetzlaff J , Altman DG , Grp P. Preferred reporting items for systematic reviews and meta-analyses: The PRISMA statement. J Clin Epidemiol. 2009;62:1006–1012. doi: 10.1016/j.jclinepi.2009.06.005. 19631508

[30] Page MJ , McKenzie JE , Bossuyt PM , et al. The PRISMA 2020 statement: An updated guideline for reporting systematic reviews. BMJ. 2021;372:n71. doi: 10.1136/bmj.n71. 33782057 PMC8005924

[31] World Health Organisation website. Number of clinical trial registrations by location, disease, phase of development, age and sex of trial participants (1999-2024) webpage, (https://www.who.int/observatories/global-observatory-on-health-research-and-development/monitoring/number-of-trial-registrations-by-year-location-disease-and-phase-of-development) Accessed January 2024.

[32] Moore TJ , Heyward J , Anderson G , Alexander GC. Variation in the estimated costs of pivotal clinical benefit trials supporting the US approval of new therapeutic agents, 2015-2017: A cross-sectional study. BMJ Open. 2020;10:e038863. doi: 10.1136/bmjopen-2020-038863. PMC7295430 32532786

[33] Unger JM , Vaidya R , Hershman DL , et al. Systematic review and meta-analysis of the magnitude of structural, clinical, and physician and patient barriers to cancer clinical trial participation. J Natl Cancer Inst. 2019;111:245–255. doi: 10.1093/jnci/djy221. 30856272 PMC6410951

[34] Meropol NJ , Buzaglo JS , Millard J , et al. Barriers to clinical trial participation as perceived by oncologists and patients. J Natl Compr Canc Netw. 2007;5:655–664. doi: 10.6004/jnccn.2007.0067. 17927923

[35] International Conference on Harmonisation (ICH) Guideline for Good Clinical Practice website, (https://www.ich.org/page/ich-guidelines) Accessed 20 January 2024.

[36] Australian Government, Department of Health and Aged Care, Therapeutic Goods Administration website. Department of health and aged care, therapeutic goods administration website. ICH guideline for good clinical practice and national statement webpage, (https://www.tga.gov.au/resources/publication/publications/ich-guideline-good-clinical-practice) Accessed 20 January 2024.

[37] Australian Clinical Trials website. Good Clinical Practice (GCP) Statement and National Statement webpage, (https://www.australianclinicaltrials.gov.au/researchers/good-clinical-practice) Accessed 20 January 2024.

[38] World Medical Association website. Declaration of Helsinki – ethical principles for medical research involving human subjects webpage, (https://www.wma.net/policies-post/wma-declaration-of-helsinki-ethical-principles-for-medical-research-involving-human-subjects) Accessed 20 January 2024.

[39] Hansen HH , Bjerre-Jepsen M , Hossfeld D. The European Society for Medical Oncology (ESMO) and its activities through the Central Eastern European Task Force. Ann Oncol. 1999;10:9–13. 10676547

[40] Yang W , Guan L. Bridging the US and China together to conquer cancer: Report of the 4th annual meeting of the US Chinese Anti-Cancer Association (USCACA). Chin J Cancer. 2012;31:315–318. 22739264 10.5732/cjc.012.10163 PMC3777500

[41] Abbott LS , Pater JL , Friel KT , Kato D , Dancey J. Improving cancer clinical trial accrual in Ontario, Canada: The Clinical Trials Infrastructure Fund (CTIF) experience. J Clin Oncol. 2014;32:6602–6602. doi: 10.1200/jco.2014.32.15_suppl.6602.

[42] Brower V. American Society Of Clinical Oncology developing first clinical trial. J Natl Cancer Inst. 2015;107:3–4. United Kingdom Oxford University Press. doi: 10.1093/jnci/djv356. 26538625

[43] Beck L , Witt R , Nesper-Brock M , et al. A study of regulatory challenges of pediatric oncology phase I/II trial submissions and guidance on protocol development. Clin Pharmacol Therapeutics. 2021;110:1025–1037. doi: 10.1002/cpt.2319. 34050933

[44] Black D , Konneh M , Sykes A. Innovative early clinical trial designs and development strategies: Evolution or revolution? highlights from the society for medicines research symposium. Drug Future. 2021;46:587–590. doi: 10.1358/dof.2021.46.7.3325410.

[45] Olawepo JO , Ezeanolue EE , Ekenna A , et al. Building a national framework for multicentre research and clinical trials: Experience from the Nigeria implementation science alliance. BMJ Glob Health. 2022;7:e008241. doi:10.1136/bmjgh-2021-008241. PMC9024272 35450861

[46] Coory MD , Ho T , Jordan SJ. Australia is continuing to make progress against cancer, but the regional and remote disadvantage remains. Med J Aust. 2013;199:605–608. doi: 10.5694/mja13.10055. Erratum in: *Med J Aust*. 2013 Dec 16;199(11):757. 24182226

[47] Muthusamy A , Long D , Underhill CR. Improving recruitment to clinical trials for regional and rural cancer patients through a regionally based clinical trials network. Med J Aust. 2021;214:453–454.e1. 33990964 10.5694/mja2.51078

[48] Sabesan S , Larkin S , Evans R , et al. Telemedicine for rural cancer care in North Queensland: Bringing cancer care home. Aust J Rural Health. 2012;20:259–264. 22998200 10.1111/j.1440-1584.2012.01299.x

[49] Sabesan S , Simcox K , Marr I. Medical oncology clinics through videoconferencing: an acceptable telehealth model for rural patients and health workers. Intern Med J. 2012;42:780–785. 21627743 10.1111/j.1445-5994.2011.02537.x

[50] Thaker D , Monypenny R , Olver I , Sabesan S. Cost savings from a telemedicine model of care in Northern Queensland, Australia. Med J Australia. 2013;199:414–417. 24033216 10.5694/mja12.11781

[51] Sabesan S , Allen DT , Caldwell P , et al. Royal Australasian College of Physicians Telehealth Working Group. Practical aspects of telehealth: Establishing Telehealth in an Institution. Intern Med J. 2014;44:202–205. 24528818 10.1111/imj.12339

[52] Sabesan S. Cancer care through telehealth models. Aust J Rural Health. 2015;23:19–23. 25689379 10.1111/ajr.12170

[53] Sabesan S , Zalcberg J. Access to clinical trials closer to home using tele-health (Chapter 10). In: Prostran M , ed. Clinical Trials in Vulnerable Populations. InTechOpen, 2018:157–175. doi: 10.5772/intechopen.70205.

[54] Clinical Oncological Society of Australia website. COSA Tele Trials Model Report 2021, (https://cosa.org.au/media/332811/cosa-tele-trials-model-report-2021-web-final.pdf) Accessed 20 January 2024. See page 5, section 4.1 Global pandemic.

[55] Pothuri B , Secord A , Armstrong A , et al. Anti-cancer therapy and clinical trial considerations for gynecologic oncology patients during the COVID-19 pandemic crisis. Gynecol Oncol. 2020;158:16–24. doi: 10.1016/j.ygyno.2020.04.694. 32386911 PMC7177100

[56] De B , Kaiser KW , Ludmir EB , et al. Radiotherapy clinical trial enrollment during the COVID-19 pandemic. Philadelphia, Pennsylvania Taylor & Francis Ltd. 2021;60:312–315. 10.1080/0284186X.2020.1865564 33356801

[57] Rubio-San-Simón A , Verdú-Amorós J , Hladun R , et al. Challenges in early phase clinical trials for childhood cancer during the COVID-19 pandemic: A report from the new agents group of the Spanish Society of Paediatric Haematology and Oncology (SEHOP). Clin Transl Oncol. 2021;23:183–189. doi: 10.1007/s12094-020-02399-3. 32472454 PMC7258607

