# Supplementary material for: Factors influencing the performance of clinical research networks to improve the success of cancer clinical trials: A scoping review and organizational analysis
Source: J Clin Transl Sci. 2025 Dec 17;10(1):e9. doi: 10.1017/cts.2025.10210 (PMC12895488; doi:10.1017/cts.2025.10210)
Supplement: Paton et al. supplementary material [file S2059866125102100sup001.docx]

**Supplementary Materials**

Appendix A: PROSPERO Protocol CRD42023414241 Search Strategy

PROSPERO Protocol CRD42023414241 was registered on 12th April 2023.

The search strategy outlined included three terms; as outlined in the table below:

1. clinical research network synonyms (Title; Abstract)
2. clinical trial synonyms (Medical Subject Headings (MeSH); key word)
3. oncology synonyms (MeSH)

| 1. | clinical trial network.tw. |
| --- | --- |
| 2. | research network.tw. |
| 3. | consortium.tw. |
| 4. | society.tw. |
| 5. | (national* adj3 institution*).tw. |
| 6. | (health* adj3 institution*).tw. |
| 7. | (national* adj3 collaborat*).tw. |
| 8. | 1 or 2 or 3 or 4 or 5 or 6 or 7 |
| 9. | clinical trial.tw. |
| 10. | "randomi?ed control* trial*".tw. |
| 11. | “clinical trial (MeSH topic)”/ |
| 12. | "randomized controlled trial (MeSH topic)"/ |
| 13. | 9 or 10 or 11 or 12 |
| 14. | “psycho-oncology/ or surgical oncology/ or oncology/ or radiation oncology” (MeSH topics)/ |
| 15. | 8 and 13 and 14 |

Abbreviations: MeSH: Medical Subject Headings;

**Supplementary Materials:**

Figure 1: PRISMA Flow Diagram

Studies from databases/registers **(n = 1928)**

Embase (n = 915)

PubMed (n = 386)

Scopus (n = 352)

MEDLINE (n = 146)

CINAHL (n = 112)

CENTRAL (n = 16)

Unspecified (n = 1)

**Identification**

References removed **(n = 662)**

Duplicates identified manually (n = 8)

Duplicates identified by Covidence (n = 654)

Studies excluded **(n = 1126)**

Studies screened **(n = 1266)**

**Screening**

Studies not retrieved **(n = 0)**

Studies sought for retrieval **(n = 140)**

Studies excluded **(n = 127)**

Not in the English language (n = 8)

Does not meet the intervention definition (n = 1)

Does not meet the network definition (n = 14)

Does not meet the original research definition (n = 18)

Does not meet the organisation systems or interventions definitions (n = 21)

Does not meet the measurement definition concerning network functionality and research optimisation (n = 35)

Reviews, guidelines, statements, awards, presidential addresses, annual reports (eg. highlights, choosing wisely, hot topics), opinion pieces, viewpoints, editorial letters, newsletter articles; abstracts, grey literature (n = 30)

Studies assessed for eligibility **(n = 140)**

**Included**

Studies included in review **(n = 13)**

**Supplementary Materials:**

Table 1 – Describes the structured eligibility criteria framework in detail in line with the PICOS framework (Richardson, 1995).

| Eligibility criteria | |
| --- | --- |
| **POPULATION** | |
| Includes | Excludes |
| - Articles related to oncology patients, clinical research and treatments.  - Articles about CRNs, with consideration to:   1. Unrestricted geographic location; 2. Constitutionally-enshrined membership framework; 3. Relevant partnerships with academic consortia. | - Articles containing individual clinical trial results that are sponsored by CRNs or single institution research projects;  - Articles about integrative therapies (eg. natural and complementary medicine) and non-human research;  - Articles about clinical research sponsored by commercial sponsors (eg. pharmaceutical companies and / or device manufacturers). |
| **Intervention / Exposure** | |
| Include | Exclude |
| - Articles evaluating the organisational characteristics, qualities and functions that influence the conduct of clinical research by the CRN. | - Articles evaluating aspects of the organisational activity of the CRN but unrelated to the conduct of clinical research. |
| **Comparator / Context** | |
| Include | Exclude |
| *- Intentionally blank* | - *Intentionally blank* |
| **Outcome** | |
| Include | Exclude |
| - Articles reporting research on the factors influencing  CRN’s performance, impact and (enabling / barrier) characteristics in relation to conducting clinical research. | - Articles reporting on measures unrelated to the CRN’s performance and impact in relation to conducting clinical research. |
| **Study Characteristics** |  |
| Include | Exclude |
| - Peer-reviewed articles that include original (qualitative, quantitative, mixed methods) research (eg. surveys, descriptive, applied research, case reports, case series, cohort studies and case control studies) from any phase or study design, reporting structured methods and results. | - Articles that include reviews, editorials, letters, treatment consensus guidelines, conference abstracts and addresses, grey literature and that are not peer-reviewed. |
| **Other** |  |
| Include | Exclude |
| - Full-text articles that are available in English. | - Articles that include full-text in any other language than English. |

Abbreviations: CRNs: Clinical Research Networks.

**Supplementary Materials:**

Table 2 – Details of recently established CRNs that formed by merging cancer CRNs that no longer exist

Of the 11 cancer CRN groups identified in this study, 3 cancer CRNs were relatively recently established (ie. NRG Oncology, Alliance and ECOG-ACRIN), emerging from member-based networks that no longer exist. All are cancer CRNs operating in the USA, North America. Descriptions of the newly formed entities, the original name of the organisations (pre-merger) and the year of merger is summarised.

Table 2: Details of recently established CRNs that formed by merging cancer CRNs (that no longer exist)

| **#** | **Name of recently established cancer CRN** | **Initial name of organisations involved (pre-merger)** | **Year of merger** |
| --- | --- | --- | --- |
| 1 | NRG Oncology Group† | National Surgical Adjuvant Breast and Bowel Project (NSABP), the Radiation Therapy Oncology Group (RTOG), and the Gynaecologic Oncology Group (GOG)) | 2014 |
| 2 | The Alliance for Clinical Trials in Oncology† | The American College of Surgeons Oncology Group (ACOSOG), Cancer and Leukemia Group B (CALGB), and North Central Cancer Treatment Group (NCCTG)) | 2011 |
| 3 | ECOG-ACRIN Cancer Research Group† | The Eastern Cooperative Oncology Group (ECOG) and the American College of Radiology Imaging Network (ACRIN) | 2012 |

Key and abbreviations:

CRNs: Clinical Research Networks; NSABP: National Surgical Adjuvant Breast and Bowel Project; RTOG: The Radiation Therapy Oncology Group; GOG: The Gynaecologic Oncology Group; ACOSOG: The American College of Surgeons Oncology Group’ CALGB: Cancer and Leukemia Group B; NCCTG: The North Central Cancer Treatment Group; ECOG: The Eastern Cooperative Oncology Group; ACRIN: The American College of Radiology Imaging Network.

† These CRNs recently formed as a result of a merging of pre-existing member-based CRNs (that no longer exist). To reflect contemporary CRN activity only the more recently established entities were analysed for this study.

**Supplementary Materials:**

Table 3 - Organisations that contributed to the eligible articles but are not cancer CRNs

Three organisations that are not cancer CRNs were excluded from the analysis.

Table 3: Organisations that contributed to the eligible studies but are not cancer CRNs

|  | **#** | **Name of organisation** | **Reason for exclusion** |
| --- | --- | --- | --- |
|  | 1 | Association of American Cancer Institutes | Does not meet the CRN definition. |
|  | 2 | Friends of Cancer Research | Does not meet the CRN definition. |
|  | 3 | The Indian Council of Medical Research | Does not meet the CRN definition. |

Abbreviations:

CRNs: Clinical Research Networks.

**Supplementary Materials:**

Table 4 – Summary of the screened but ineligible articles that referred to a cancer CRN

During screening, 25 cancer CRNs were identified in articles that were ineligible for inclusion. Of these, 24 CRN are operating and 1 CRN is in the process of being dismantled (ie. National Cancer Research Institute in the United Kingdom).

Table 4: Summary of the screened but ineligible studies that referred to a cancer CRN

| **Name of cancer CRN**  **Location (continent, headquarter country)** | **Acronym** |
| --- | --- |
| ASIA-PACIFIC | |
| Australia | |
| Trans-Tasman Radiation Oncology Group | TROG |
| China |  |
| Chinese Society of Clinical Oncology | CSCO |
| Japan |  |
| Cancer Genomic Medicine Japan | CGM Japan |
| Japanese Clinical Oncology Group | JCOG |
| Japan Society for Cancer Therapy | JSCT |
| Japanese Society of Haematology | JSOH |
| West Japan Lung Cancer Group | UNK |
| South Korea |  |
| Korean Society of Paediatric Neuro-Oncology | KSPNO |
| EUROPE | |
| Brussels |  |
| European Research and Treatment of Cancer | EORTC |
| European Society of Medical Oncology | ESMO |
| Germany |  |
| Paediatric Oncology and Haematology in Germany | GPOH |
| Spain |  |
| Spanish Society of Paediatric Haematology and Oncology Group* | SEHOP |
| Switzerland |  |
| European Thoracic Oncology Platform | ETOP |
| International Society of Paediatric Oncology and subgroup | SIOP |
| NORTH AMERICA | |
| Canada |  |
| Canadian Cooperative Trials Group | CCTG |
| United States of America |  |
| American Society of Oncology*, and various subgroups and research consortiums | ASCO |
| American Society for Therapeutic Radiology and Oncology | ASTRO |
| Association of American Cancer Institutes* | AACI |
| Childrens' Oncology Group Paediatric Brain Tumor Consortium | COG PBTC |
| Chinese American Citizens Alliance | CACA |
| Formerly the Multiple Myeloma Research Consortium, currently the Multiple Myeloma Research Foundation | Formerly MMRC, currently MMRF |
| National Cancer Institute* Cancer Research Network, evolved in to the National Clinical Trials Network in 2014; subcommittee Early Detection Research Network | Formerly NCI CRN, currently NCI CTCN; EDRN |
| Society of Gynaecologic Oncology | SGO |
| Society of Surgical Oncology | SSO |
| United Kingdom | |
| Formerly National Cancer Research Network, UK, currently National Cancer Research Institute, UK# | Formerly NCRN, currently NCRI |

Key and abbreviations:

CRNs: Clinical Research Networks; USA: United States of America; UK: United Kingdom.

* These organisations also contributed some research that met the PROSPERO protocol eligibility criteria and these articles were included and analysed in this study.

# An announcement confirming that the NCRI is being dismantled was made in 2023, see: https://www.ncri.org.uk/ncri-is-winding-down-after-22-remarkable-years/.

**Supplementary Materials:**

Table 5: List of locations (continent, headquarter country) for screened but excluded articles.

Researchers from cancer CRNs in 12 countries and 3 continents (self-described in their publications) contributed research but their studies were ineligible for inclusion in this study.

Table 5: List of locations (continent, headquarter country) for screened but excluded articles.

| **Location (Continent, Headquarter Country)** |
| --- |
| AFRICA |
| Nigeria |
| ASIA |
| China |
| Japan |
| AUSTRALIA |
| Australia |
| EUROPE |
| Germany |
| Holland |
| Italy |
| Malta |
| Spain* |
| Russia |
| NORTH AMERICA |
| USA* |
| Canada |
| CONTINENT (self-described in manuscript) |
| Africa* |
| Asia |
| Central and Eastern Europe |

Key:

* Researchers in these locations also contributed research that was screened and included (see Main Paper Table 5), however there were more items from other researchers in these same locations that were screened and excluded; for these reasons the location has been retained in this table.

**Supplementary Materials:**

Table 6: Excluded articles

A list of studies excluded at the full-text screening level (sorted by reason for exclusion) are available as supplemental material. Extraction tables can be provided on request.

**Supplementary Materials:**

Table 7: Characteristics of cancer CRN organisation involved in each of the selected studies; including the service provision arrangements supporting the phase I-IV IIT clinical research capacity.

| **Name of CRN [Acronym]** | **Does the CRN have publicly available policies, infrastructure, processes to support trial sponsorship and research collaboration?** | **Does the CRN sponsor trials?** | **What types of clinical research are developed by the CRN?**** |
| --- | --- | --- | --- |
| International Society of Geriatric Oncology [SIOG] | Yes | No | NA |
| American Society of Clinical Oncology [ASCO] | Yes | Yes | I-IV |
| Children’s Oncology Group [COG] | Yes | Yes | I-IV |
| Spanish Society of Paediatric Haematology and Oncology [SEHOP] | No | No | Oth (registries only) |
| Society of Neuro-Oncology [SNO] | Yes | Yes | I-IV |
| African Organisation for Research and Training in Cancer [AORTIC] | Yes | No | NA |
| NRG Oncology Group † [NRG Oncology] | Yes | Yes | I-IV |
| The Alliance for Clinical Trials in Oncology † [Alliance] | Yes | Yes | I-IV |
| ECOG-ACRIN Cancer Research Group † {ECOG-ACRIN] | Yes | Yes | I-IV |
| The South Western Oncology Group [SWOG] | Yes | Yes | I-IV |
| National Cancer Institute [NCI] | Yes | Yes | I-IV |

Key and abbreviations:

CRN: Cancer Research Network; NA: Not applicable; I-IV: Phase I-IV clinical trials; Oth: Other.

| **What types of clinical research are developed by the CRN? Specify: I-IV: Phase I to IV clinical trials, Oth: Other (specify), NA: Not applicable (ie. the CRN does not sponsor trials and does not develop trials). |
| --- |

**Supplementary Materials:**

Table 8: A description of the associated characteristics of each study, informing the identification and definition of factors influencing the performance of each CRN to conduct their cancer clinical trials.

| **Study** | **Associated characteristics reported by each study** | | **The characteristics was evaluated to identify factors for each study** |
| --- | --- | --- | --- |
|  | **Enabling characteristics** | **Barrier characteristics** | **Factors** |
| Monfardini (2007) | ・Dedicated clinics for the target population. ・MDT case conferences meetings. ・Known referral pathways. ・Opportunity to join trials. ・Availability to training and education. ・Adequate time and resources. ・Type of practice (eg. primary care vs tertiary hospital). | ・High variability of research activity in different places. ・Absence of a CRN for professional network to support research. ・Need for future resources to support more training, resources and research. ・Trial access outside of cancer hospitals. ・Aging patients perceived as more difficult to enrol in trials (ie. they may present with comorbidities, have a reduced tolerance for chemotherapy, heterogeneity of patients, lack of clinical study background (eg. health literacy, family influence, financial problems, referral bias, lack of social network, absence of formal care givers). ・Lack of dedicated trials for older people (aged 70 years or older). ・Absence of standard definitions and absence of treatment guidelines. ・Clinical trials perceived as time consuming and there is a lack of funding for IIT studies. ・Absence of dedicated department for speciality (ie. geriatric oncology). | ・CRN ・Site ・Patient ・Policy |
| Denicoff (2013) | **Enabling characteristics** | **Barrier characteristics** | **Factors** |
|  | ・Different strategies are needed for the different learning styles of potential study participants, as well as the unique needs of different types of studies (eg. protocol research questions and study design). ・Patient / community recommendations: demonstrated, meaningful health consumer engagement across the trial life cycle. ・Appropriate use of screening logs, simplification of documents to help improve health literacy. ・Improved trial awareness (websites, videos, registries, commercial marketing approaches, personal connection, improved health literacy, simplified consent forms, decision aids). ・Physician / provider recommendations: ・Institutional commitment and senior leadership are important. ・Access to dedicated research teams, back by MDT approaches. ・Improved training and Continuing Professional Development (CPD) accreditation processes. ・Standardised costs and financial information may enhance informed consent discussions. ・Tertiary centres are better set up for trials, and track record. ・Access to MDT. ・Past Chief Investigator (CI) experience running trials. ・Protocol development (to include recruitment planning). ・Site / organisation recommendations which recognise the importance of culture and leadership. ・Consider applicability of Master of Business Administration (MBA) style metrics to help clinical leaders perform better. ・Application of site benchmarks (expected vs actual), resourcing (staff Full Time Equivalent (FTE) + workload), number and complexity of trials, impact of technology (eg. data management software), use of screening logs. | ・Lack of published, methodologically rigorous research to improve accrual. The few studies that exist demonstrate that no one single recruitment strategy was successful across all sites / different trials. ・Reviews have noted that either methodological limitations of other studies prevent evidence-based conclusions being drawn and that there is a lack of evidence that many of the interventions did in fact lead to higher accrual. | ・CRN ・Site ・Patient ・Policy |
| Dandekar (2016) | **Enabling characteristics** | **Barrier characteristics** | **Factors** |
|  | ・Protected time for research at institutions. ・Increasing manpower to help doctors prioritise their time. ・Training in clinical trials. | ・ Lack of funds. ・ Lack of training. ・ Lack of time. ・Inability to maintain follow up of subjects. ・Lack of encouragement. ・Need for better databases to help with patient follow up. | ・CRN ・Site |
| Vose (2016) | **Enabling characteristics** | **Barrier characteristics** | **Factors** |
|  | ・Need for master templates, agreements, financial models. ・Need to ensure good legal access. ・Need for pragmatism and real-world application. ・Need to apply metrics to standardise negotiations, consider incentivisation to use existing contract templates. ・Need to clearly identify stakeholders. ・Need to contract efficiently and be sensible. ・Need to identify a value proposition to external providers to justify involvement to support trials, educate stakeholders so that they have common understanding about protocols + services. ・Address cultural disconnect between lawyers, researchers and sponsors. ・Need for greater efficiencies for site billing (software as well as site processes). ・Need for more site training on trials and management systems and estimate versus actual costs. ・Harmonise training and guidelines nationally set up a database (eg. electronic Health record equivalent). ・Centralising documents simplifies site and sponsor requirements. | ・Need for streamlined site, sponsor and regulatory administration (eg. contract negotiations with sponsor, contract negotiations with CRO, Site compliance with sponsor / CRO requirements). ・Resourcing for site monitoring visits, management of regulatory documents and AE / Serious AE (SAE) reporting. ・Resourcing for sites to attend to sponsor data management queries, close out requirements and long-term patient follow up. ・Inadequate site staffing to handle regulatory burden faced by sites. ・General lack of (site) awareness to trial accrual tools developed by national CRNs and equivalent. ・Governance issues and concerns about bureaucracy. ・Overcoming challenges with clinical trial contracts, sponsor, CROs + interinstitutional contracts (related to not limited to intellectual property, confidentiality, lack of transparency for inter-institutional protocol assessments). ・Time-intense site study feasibility pre-selection. ・Need for improvement in site clinical trial financial modelling + reimbursement (from government, insurers, hospitals, service providers). ・Need for site compliance with regulatory and training requirements (ie. inefficient need to have Good Clinical Practice (GCP) training every time, inefficiencies re. AE / SAE reporting expectation; for SUSARs there is poor education about the minimum data sets and this is a big burden on sites). ・Auditing and monitoring takes up a lot of site time, effort and expense. | ・CRN ・Site ・Regulatory |
| Szczepanek (2017) | **Enabling characteristics** | **Barrier characteristics** | **Factors** |
|  | ・Need to simply administrative burden at a site level will ensure trials are available more rapidly for patients. ・ Harmonising sites in the USA as well as research protocols (and other study docs). ・Clarity transparency and consistency for financial modelling during the study design phase, as well as during study conduct. ・Develop templates and central processes, these things can help to develop best practice. | ・Coverage analysis was identified as a burden that impacted trial participation. ・Lack of resources and expertise of coverage analysis exist, lack of Medicare coverage standard of care treatments (SOC) versus research costs, lack of staffing and time. ・ Suggestions made for more training, resources, consultations, expertise, financial support, time training, infrastructure, centralised processes and mechanisms. ・Gaps and needs re coverage analyses include: process and procedure mapping, resources, education, training, buy in, communications and feedback mechanisms, prioritisation of studies, establishing success metrics – (eg. stakeholder, sponsor and site satisfaction, impact of local research site workload). ・There is a need for improved communication between all stakeholders. | ・Site ・Patient ・Policy |
| Kim (2017) | **Enabling characteristics** | **Barrier characteristics** | **Factors** |
|  | ・Need to modernise eligibility criteria and changing clinical trial designs to maximise patient participation in trials (re. brain metastases, Adolescent and Young Adult (AYA) populations, patients with HIV, organ dysfunction and prior and current malignancies). ・Need to enrich trials with broader patient populations, analyse separately and together, accommodate additional monitoring as required. ・ Improving these things may allow for broader drug listings at registrations. | ・Broadening criteria may create safety risks which mean more screening and monitoring may be required. ・Suggestion to adjust protocol to make these things better managed. ・Need to educate IRBs and scientific committees to educate them on the need for inclusive trial design may help overcome concerns from oversight bodies. | ・CRN ・Site ・Patient ・Regulatory |
| Waterhouse (2020) | **Enabling characteristics** | **Barrier characteristics** | **Factors** |
|  | ・Opportunities to improve trial function, telehealth, remote patient monitoring, remote site initiation and monitoring by sponsors and Clinical Research Organisations (CROs), remote safety laboratory collection, improved efficiencies during study start up and conduct (eg. electronic ethics communications, remote consent), greater flexibility with drug and specimen distribution, management and documentation, streamlined data collection, reduction in collection of “unnecessary data”. ・Increased remote work may also bolster workface to improve job productivity, satisfaction, staff retention and mitigate space issues at site. ・ Opportunity for more pragmatic trials which would be beneficial to patients and research programs. ・Need to apply a centralised, risk-based approach to monitoring (in line with the guidelines) may suffice. ・Administrative suggestions to protocols and study processes (eg. use of e-signatures should become standard practice, leverage the use of e-consent.; minimisation of patient visits). ・Post-COVID 19 learnings: There is a need to develop formal policies to manage COVID, and these could be repurposed for other crisis; Improve communications at site (eg. work huddles); promote remote patient monitoring; implement remote symptom and Adverse Event (AE) review (eg. phone); prioritise clinical trial resources proactively (which trials should have screening or not); promote remote initiation and monitoring visits by sponsors and CROs; use remote lab collections where feasible; ship study investigational products directly to patients; regularly communicate with IRBs/ethics as needed; ensure appropriate and thorough documentation to processes (eg. marking “COVID” as needed; consider role of telehealth visits; cessation of ‘research only’ study visit to prioritise Standard of Care (SOC) visits; Minimise research blood or tissue collection; Need to prioritise enrolment for certain clinical trials, risk-based approach and prioritisation mindful of patient needs, safety, and disease severity, potential patient and site burdens, availability and allocation of program resources. | ・Decrease in patient willingness or ability to attend sites, effort to engage in telehealth, limited access to ancillary services. ・Time spent with sponsors, IRBs, CROs modifying trial protocol procedures is challenging, need to minimise duplication of requests from sponsors and CROs has been confusing. | ・CRN ・Site ・Patient ・Regulatory |
| Roth (2020) | **Enabling characteristics** | **Barrier characteristics** | **Factors** |
|  | ・Opportunity for MDT tumour boards. ・Establishment of site specific “AYA Champions”. ・Need to support and expand the AYA network within the CRN. | ・Lack of available trials. ・Poor communications between paediatric and medical oncology. ・Logistical constraints of accessing trials (institutional challenges between small and tertiary centres, human research ethics committees, Medicare, insurance challenges re $ who pays for what is AYA and does AYA count?). ・Need for leadership support, sufficient resources and appropriate policies (development of AYA specific support to boost activity and trials, increasing flexibility around age ranges to access inpatient and outpatient care, need to allocate space at the site). | ・CRN ・Site ・Regulatory |
| Rubio San Simon (2021) | **Enabling characteristics** | **Barrier characteristics** | **Factors** |
|  | ・Strong increase in paediatric trials due to new legal framework (eg. 2006 European Paediatric Regulations encouraged pharmaceutical companies to accelerate childhood cancers and the absolute number of trials and marketing authorisations increased after 2007). ・Increase in collaboration and involvement in international research networks. ・MDT cooperation between academia, regulators, advocacy groups and commercial pharmaceutical companies. ・Collaborations between and across hospitals and global networks helped increase trial activity and overall performance. ・More recent increase in molecular medicine early phase clinical trial activity. ・There is need to have faster start up times and regulatory approval times. | ・There are financial limitations within institutions (sites). ・There is significant national / global competition (sites + CRNs). ・There is a lack of clinical research personnel in hospitals. ・The need to have access to academic of commercial CROs to conduct the trials. | ・CRN ・Site ・Industry ・Regulatory |
| Bagley (2022) | **Enabling characteristics** | **Barrier characteristics** | **Factors** |
|  | ・Overly restrictive protocol, performance score assessment, inclusion / exclusion criteria (including irrelevant pre-study treatments) hinders accrual (limiting CRN, site and health consumer engagement). ・Inadequate phase II study designs, appropriate use of control arms. ・Regulatory approval conventions with consideration to new adaptive platform trial designs. ・Promotion of greater intergroup collaboration globally. ・Recommendations re. protocol study design, endpoints and statistics, infrastructure and patient accrual. | ・Lack of data hindering progress in the field. ・Overly restrictive eligibility criteria. ・Need for more real-world research approached. ・Need for flexible clinical trial protocol designs to maximise patient participation in trials, eg. inappropriate use of Performance Score limiting patient access (even though it would not affect the integrity of the protocol), inappropriate restriction for patient who may have had local treatment prior (eg. radiotherapy) which may have been considered Standard of Care (not experimental) so the need to reflect Real World Patient populations at study entry. ・ Inadequate Phase 2 trial designs (ie. insufficient data + generalisations) which lead to inadequate Phase III studies that may not meet their endpoints. ・Industry + CRNs (who are the trial sponsors) need to design trial pipelines with more rigour. | ・CRN ・Site ・Patient ・Policy ・Regulatory |
| Kizub (2022) | **Enabling characteristics** | **Barrier characteristics** | **Factors** |
|  | ・List of potential partner organisations and projects to improve cancer care and trial access in Africa.  ・List of potential partner and projects to improve clinical trial regulation frameworks and guideline harmonisation in Africa. ・There is an existing authored ‘guiding principles for expanding access to clinical trials to patients in Africa’ document which outlines the need for funding and technical support for local IITs on the basis of existing needs and resources that have the greatest potential impacts. | ・The need for better capacity building initiatives across various stakeholders relevant to cancer control in Africa (eg. study refers to institutions, health consumer advocacy groups, regulatory agencies, global pharmaceutical companies, global health Not-For-Profit groups and ASCO). ・Including but not limited to the need for adequate infrastructure, communications, sufficient financial and human resourcing (eg. current situation lacks trained staff), appropriately responsive regulatory systems, appropriate research systems and technology (and prioritisation capacity), appropriately supportive research environments including education, training, and networking opportunities; competing demands of time and resources across the stakeholder matrix. | ・CRN ・Site ・Patient ・Industry ・Regulatory |
| Higgins (2022) | **Enabling characteristics** | **Barrier characteristics** | **Factors** |
|  | ・Study Start Up: Implementation of a tool kit including: Powerpoint slides – saved on website, patient brochure, website landing page, (accessible to the general public), email template upon activation, 30 to 60 sec study chair patient / health consumer education video, video script shared on social media (eg. via institutional twitter and Facebook accounts), study kick-off session presented at the CRN’s semi-annual meeting to educate clinicians and research about protocol and improve general awareness across CRN’s membership.  ・During Trial: Regular trial updates encouraged by CRN’s Champions. Trial in Progress abstract at relevant fora (eg. ASCO). Study team are expected to conference speaking opportunities to promote the study. Industry collaborations are expected to promote the study at industry opportunities where appropriate. Use of social media is vital. Study-specific newsletters are sent monthly to study sites (this records site approval status, monthly and overall accrual performance).  ・Every trial must have a dedicated Patient Advocate Champion identified. ・Good communication is necessary across the stakeholder matrix. ・The tool kit is low cost and require modest infrastructure to implement. | ・Monthly phone calls and or webinars can be arranged between sites and the study team to boost accrual. ・Targeted communications with the community intermediaries including disease specific committees and patient / health consumer advocates. ・Monthly operations meetings with the sites (phone calls, webinars, in person) to understand the site accrual experience, patients declining the study (ie. screen fails). This may lead to study amendments or adjustments. For trials that are failing a patient-specific landing website can be created.  ・Encourage and promote study across networks, including identifying CRN champions to promote the study (if not already identified).  ・Consideration to open new sites via a feasibility process coordinated by the CRN.  ・Oversight by the CRN oversight committee can also help boost accrual for underperforming studies.  ・In this study, the CRN has a project operations management committee that reviews trial performance quarterly which helps to identify any study issues early. | ・CRN ・Site ・Patient |
| Mittal (2022) | **Enabling characteristics** | **Barrier characteristics** | **Factors** |
|  | ・Recommendation to have a trial champion, improving interdisciplinary communications between stakeholders (ie. between specialities paediatrics + medical oncology).  ・Access to site education (eg. increasing site and provider awareness of studies that may lead to decreasing barriers).  ・Getting more resources to open studies for enrolment.  ・Identifying champions at sites.  ・Additional CRA and regulatory staff support would help facilitate accrual.  ・Study suggested tumour boards, MDTs, newsletters, Standard Operating Procedures (SOPs), and less formal methods to improve communications (eg. phone calls and emails between site staff) may help accrual. ・Hosting webinar (quarterly), newsletter distribution to the study members at site may help accrual. | ・Site reasons for not opening a trial were limited research staff and resources, low anticipated accrual, lower prioritisation of the trial, high staff turnover, high burden of opening processes, lack of knowledge about the study, regulatory delays and the study being deemed a lower priority. ・Regulatory and financial burden on sites were reported when they conduct CRN studies (due to minimal resourcing).  ・Reduced prioritisation by the sites for these kinds of IIT studies (due to minimal resourcing).  ・In the USA different sponsorship arrangements may affect the site leadership (eg. AYA sponsor vs CRN sponsor vs site institutional sponsor) creating confusion and creating delays. The study suggests that trial champions at the site may resolve and address any potential confusion. | ・Site ・CRN ・Regulatory |

Abbreviations:

CRN: Clinical Research Network; MDT: Multi-Disciplinary Teams; MBA: Master of Business Administration; CPD: Continuing Professional Development; ICH-GCP: International Council for Harmonisation's Good Clinical Practice (GCP) guidelines, an international standard for ethical and scientific quality in clinical trials involving humans; GCP: Good Clinical Practice; FTE: Full time equivalent; IRB: Institutional Review Board; HREC: Human Research Ethics Committee; SOPs: Standard Operating Procedures; IT: Information Technology ; AEs: Adverse Events; SAEs: Serious Adverse Events; IP: Intellectual Property; CRO: Clinical Research Organisation; CI: Chief Investigator; SOC: Standard of Care treatments; USA: United States of America; AYA: Adolescent and Young Adult; ASCO: The American Society of Clinical Oncology; IITs: Investigator-Initiated Trials.
